# Migratory birds have a distinct haemosporidian community and are temporally decoupled from vector abundance at a stopover site

**DOI:** 10.1017/S0031182024001239

**Published:** 2024-10

**Authors:** Spencer C. Galen, Emily Ostrow, Suravi Ray, Marissa Henry, Janice Dispoto, Alison Fetterman, Lisa Kiziuk, Jason D. Weckstein

**Affiliations:** 1 Biology Department, University of Scranton, Loyola Science Center, Scranton, PA, USA; 2 Department of Ornithology, Academy of Natural Sciences of Drexel University, Philadelphia, PA, USA; 3 Department of Biodiversity, Earth, and Environmental Science, Drexel University, Philadelphia, PA, USA; 4 Southwestern Native Aquatic Resource and Recovery Center, United States Fish and Wildlife Service, Dexter, NM, USA; 5 Willistown Conservation Trust, Newtown Square, PA, USA

**Keywords:** bird, haemosporidian, migration, stopover site, vector

## Abstract

Migratory animals likely play an important role in the geographic spread of parasites. In fact, a common assumption is that parasites are potentially transmitted by migratory animals at temporary stopover sites along migratory routes, yet very few studies have assessed whether transmission at stopover sites can or does occur. We investigated the potential for a group of vector-transmitted parasites, the avian haemosporidians, to be transmitted during migratory stopover periods at Rushton Woods Preserve in Pennsylvania, USA. Using an analysis of 1454 sampled avian hosts, we found that while a core group of abundant haemosporidians was shared between local breeding birds and passing migrants, the parasite community of migratory birds at Rushton was distinct from that of local breeding birds and showed similarity to a previously sampled boreal forest haemosporidian community. Haemosporidians that were unique to passing migratory birds were associated with sampling sites in North America with cooler summer temperatures than haemosporidians that are transmitted at Rushton, suggesting that the transmission of these parasites may be restricted to high-latitude regions outside of our temperate stopover site. We also found that the abundance of mosquitoes in our study region is offset from that of migratory bird abundance during avian migratory periods, with the peak period of bird migration occurring during periods of low mosquito activity. Collectively, these findings suggest that although abundant haemosporidians are possibly transmitted between local and passing migratory birds, a combination of biotic and abiotic factors may constrain haemosporidian transmission during avian stopover at our study site.

## Graphical abstract

**Figure:**
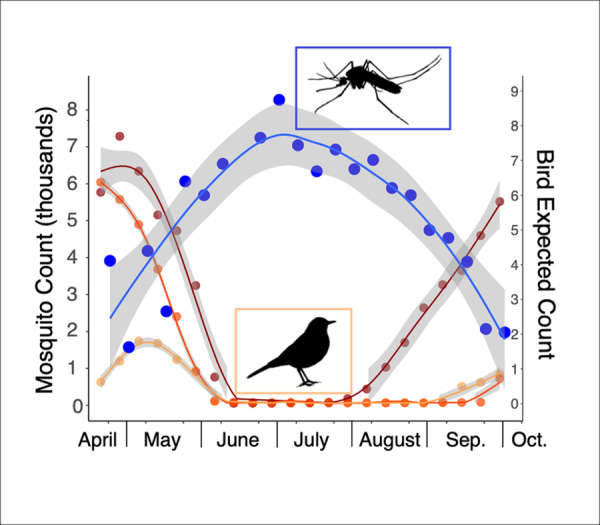

## Introduction

Animal migration and parasitism are intimately linked. From a parasite's perspective, migration of its host provides an opportunity for dispersal to new regions and new pools of potential hosts (Boulinier *et al*., 2016; Briscoe *et al*., 2022). Indeed, parasites that infect migratory host species tend to have larger geographic ranges than parasites that infect only resident host species (de Angeli Dutra *et al*., 2021*a*). Host migration has also been hypothesized to promote parasite diversification by facilitating host-switching (Jenkins *et al*., 2012), and may influence the evolution of numerous parasite traits such as virulence and lifecycle phenology (Poulin and de Angeli Dutra, 2021).

From the host perspective, migration can provide an opportunity to escape parasitism if infection prevalence or intensity is locally high year-round and can be avoided by breeding in locales geographically distant from non-breeding sites (Loehle, 1995; Balstad *et al*., 2021). However, the opposite can be true as well – some host species appear to migrate from regions of low parasite transmission to breeding locations where parasites are frequently acquired (Pulgarín-R *et al*., 2019). In general, migratory animals appear to host higher parasite species richness than non-migratory animals (Emmenegger *et al*., 2018; Poulin and de Angeli Dutra *et al*., 2021). Parasitism can also negatively impact host body condition, preventing a host from becoming physiologically prepared to endure a gruelling migration (Bradley and Altizer, 2005; Mages and Dill, 2010; but see Hahn *et al*., 2018 for an example of haemosporidian parasites having no detectable impact on avian migratory capacity). Parasitism could also potentially delay migration or impact the ability of a host to properly navigate to their destination (Møller *et al*., 2004; Santiago-Alarcon *et al*., 2013; Hegemann *et al*., 2018).

Much has been written about the potential for migratory host species to spread parasites across large geographic distances (Altizer *et al*., 2011; Fritzsche McKay and Hoye, 2016). It is clear from analyses of parasite biogeographic history that parasites can shift transmission areas over evolutionary time (Hellgren *et al*., 2007; Hoberg and Brooks, 2008), presumably by hitching a ride to new regions using migratory hosts followed by local transmission. However, a lesser-studied phenomenon is the transmission of parasites between local and migratory hosts at stopover sites between the wintering and breeding grounds where migratory animals temporarily pause their movements. Parasite transmission during migration, between passing migrants and local resident individuals, has been speculated on as a potentially important phenomenon that would give parasites numerous opportunities to spread into new communities as their hosts migrate (Figuerola and Green, 2000; Altizer *et al*., 2011; Ciloglu *et al*., 2020; de Angeli Dutra *et al*., 2021*b*). However, parasite transmission during migration has been rarely documented, and only in directly transmitted parasites (e.g. viruses in birds, Krauss *et al*., 2010; protozoans in butterflies, Satterfield *et al*., 2018). An open question is whether vector-mediated parasites are transmitted during host migration, and if so, whether this event occurs routinely (Ishtiaq and Renner, 2020).

It is highly difficult to document parasite transmission in action in a natural environment, particularly among highly active and volant hosts such as birds. As a result, the question of whether migratory hosts spread or acquire parasites at stopover locations along their routes has not been directly addressed in most systems. However, there are several ways to test whether parasite transmission between passing migrants that use stopover sites and local hosts is likely to occur without observing the acquisition of parasites in real time. First, we can ask whether the community of parasites that infects migratory hosts overlaps with the parasite community of local hosts. A homogeneous parasite community between migratory and local hosts would suggest frequent parasite transmission between hosts with different migratory strategies, whereas high turnover between them would suggest that there is some barrier to transmission between migratory and local hosts. We can also examine the parasites that are unique to migratory hosts, and test whether they are associated with a climate that is distinct from the stopover site. A parasite carried by a migratory host may not successfully transmit to a local host at a stopover site if the abiotic conditions are not favourable for transmission. Lastly, in the case of vector-borne parasites, we can assess whether the activity of known vector species corresponds with host migratory periods. A lack of temporal overlap between migrant and vector activities would provide strong evidence that transmission between migratory and local hosts is unlikely to occur during stopover.

Blood parasites of birds are an excellent group with which to evaluate transmission between migratory and local hosts at stopover sites. Migration in birds is a well-studied phenomenon, and with the advent of community science initiatives such as eBird (Sullivan *et al*., 2009) and novel technologies for tracking birds across their annual cycles (McKinnon and Love, 2018), our understanding of avian migratory routes and stopover duration has greatly accelerated in recent years. Importantly, birds are also hosts to a wide array of easily studied endo- and ectoparasites that can be tracked across the avian annual cycle (Pulgarín-R *et al*., 2019). Parasites in the order Haemosporida, which includes malaria parasites and their relatives, are one of the best-studied groups of wildlife parasites and are ubiquitous parasites of birds globally (Fecchio *et al*., 2021). The existence of the MalAvi database (Bensch *et al*., 2009), a centralized repository for avian haemosporidian infection records from tens of thousands of sampled hosts, has made this group of parasites an emerging model for disease ecology.

Although avian haemosporidians have been studied intensively within communities of breeding birds and communities of migrating birds separately, the study of haemosporidians at a single location across multiple seasons is rare (e.g. Huang *et al*., 2022) and it is even more uncommon to evaluate the potential for transmission of haemosporidians between local and migratory hosts. Here, we tested the hypothesis that haemosporidian transmission between local and migratory birds is infrequent and is limited by climate and vector abundance. We tested this hypothesis by characterizing the haemosporidian communities of local and migratory birds, as well as migratory bird and vector abundances at a temperate stopover site in eastern North America.

## Materials and methods

### Study site and sample collection

Samples were collected at Rushton Woods Preserve (hereafter referred to as ‘Rushton’), which is 86 acres of protected temperate forest and fields in Newtown Square, Chester County, Pennsylvania (39.984°N, −75.486°W). Sampling occurred between 12 April and 4 November in 2015, 2016 and 2018, and from 22 May to 31 July in 2019. Bird sampling was performed in accordance with the Drexel University Institutional Animal Care and Use Committee under protocol no. 20689 and the USFWS banding permit no. 23679. Birds were captured using mist nets and banded with uniquely numbered USFWS bands. We collected up to 70 μL of blood from each bird by puncturing the brachial vein with a 29-gauge needle. We used this blood to make 2 blood smears on microslides and then froze the remaining blood in liquid nitrogen to preserve DNA. The blood smears were air dried and fixed in methanol. Upon returning to the lab, the slides were stained for 50 min using a 10% Giemsa stain, databased and deposited in the Academy of Natural Sciences of Drexel University bird blood film collection. The collected blood smears were not examined for the current study.

We sought to compare haemosporidian communities between avian hosts that breed at Rushton (‘local breeding birds’) and those that use Rushton temporarily during their annual migrations (‘passing migrants’). We further classified local breeding birds as ‘residents’ that are not known to exhibit migratory behaviour, and migratory species that reproduce at Rushton (‘breeding migrants’). In contrast, passing migrants were classified as either migratory species that are not known to reproduce at Rushton (‘complete passing migrants’), or individuals of migratory species that are known to breed at Rushton, but were using Rushton as a stopover site at the time of sampling (‘non-breeding migrants’). To distinguish between ‘breeding migrants’ and ‘non-breeding migrants’, which can consist of individuals from the same bird species, we used a combination of date cutoffs, evidence of reproductive behaviour (e.g. cloacal protuberance or brood patch) and evidence of site fidelity from banding records. A detailed description of the data sources that we used to make this distinction is given in the Supplementary Methods.

### Molecular methods

DNA was extracted from the blood samples using the Qiagen DNeasy 96 Blood & Tissue Kit (Qiagen, Germantown, MD, USA) protocol. Each sample was screened for avian haemosporidian parasites of the genera *Plasmodium*, *Haemoproteus* (including the subgenus *Parahaemoproteus*) and *Leucocytozoon* using a standard cytochrome b (*cytb*) barcoding approach following Carlson *et al*. *(*2018). The resulting consensus sequences were used to identify unique haemosporidian *cytb* haplotypes, which we refer to as ‘genetic lineages’ following the standard in avian haemosporidian research (Bensch *et al*., 2009). The resulting consensus sequences were either assigned to genetic lineages already present in the MalAvi database (Bensch *et al*., 2009), or were given new lineage names using MalAvi naming standards (first 3 letters of the host genus and species followed by a unique number). Chromatograms with overlapping traces in at least 1 nucleotide position were identified as mixed infections with multiple haemosporidian lineages and parsed apart manually following Starkloff and Galen (2023).

As we were interested in species-level differences in haemosporidian communities, we used the following framework to identify lineages that reflect putative haemosporidian species (rather than intraspecific *cytb* variants that would inflate community alpha diversity). Any rare (5 or fewer occurrences) lineage that differed from a more abundant lineage by just 1 base pair was ‘lumped’ into the abundant lineage for analysis. This decision was justified by previous studies showing that haemosporidian lineages that differ by 1 base pair often share nuclear genotypes (*Haemoproteus*: Bensch *et al*., 2004; *Leucocytozoon*: Galen *et al*., 2018; *Plasmodium*: Hellgren *et al*., 2015).

We identified haemosporidian lineages that are transmitted in North America as those sampled either in host species that do not exhibit migratory behaviour, or in hatch-year birds that had never left North America at the time that they were sampled. Haemosporidians were determined to be transmitted at Rushton specifically if the parasite was found to infect resident host species or hatch-year individuals of migratory species that breed at Rushton and were sampled between 1 June and 10 August.

### Phylogenetic analyses

Using BEAST2 (Bouckaert *et al*., 2014), we estimated a Bayesian phylogeny for all haemosporidian *cytb* lineages that we detected. We identified the best-fit model of evolution as GTR + I + G using jModelTest2 (Darriba *et al*., 2012), and implemented a strict clock and a Yule speciation model. We ran the analysis for 10 million generations, sampling every 1000 generations. We summarized the posterior distribution of topologies using TreeAnnotator, discarding the first 10% as burn-in to estimate the maximum clade credibility tree. For avian host species, a distribution of 100 phylogenies for all host species that were sampled was downloaded from www.birdtree.org (Jetz *et al*., 2012) using the ‘Ericson all species’ option. A majority clade credibility tree was estimated from this distribution using TreeFinder.

### Haemosporidian beta diversity and ecological traits

We characterized the beta diversity of haemosporidian communities infecting local breeding birds and passing migrants at Rushton using R (version 4.1.1, R Core Team, 2022) and the package *betapart* (Baselga and Orme, 2012) with the function ‘beta.pair’ to estimate total beta diversity and its turnover (replacement of species) and nestedness (the degree to which 1 community is a subset of another) components using the Jaccard index. We also measured haemosporidian beta diversity among hosts in different migratory categories using the unweighted form of the UniFrac distance (Lozupone and Knight, 2005), which we used to incorporate the phylogenetic distances among parasite lineages within communities. We calculated UniFrac distances using the ‘UniFrac’ function in the *phyloseq* package (McMurdie and Holmes, 2013). To provide more geographic context to the comparison of the haemosporidian communities of birds of different migratory categories at Rushton, we also tested whether the haemosporidian community of passing migrants was more similar to birds that breed at Rushton than to haemosporidians from high-latitude bird communities in North America. For this analysis, we used the previously described beta diversity methods (Jaccard index and UniFrac distance) to compare the data from Rushton to community data from a survey of avian haemosporidians in breeding birds in 6 sites in central Alaska (Galen *et al*., 2019), which is one of the only community-wide survey of avian haemosporidians from a high-latitude location in North America.

In addition, we tested whether local breeding birds and passing migrants at Rushton had significantly different haemosporidian communities. We constructed a host–parasite interaction matrix with hosts as the rows and parasites as the columns, and used a binary variable of ‘host breeds at Rushton’ and ‘host uses Rushton as a stopover site’ as the independent variable. We used the ‘adonis2’ function in the *vegan* package (Oksanen *et al*., 2022) to conduct a permutational analysis of variance (PERMANOVA) using 999 permutations. We conducted PERMANOVA using 3 beta diversity metrics: the Jaccard index, which uses binary presence/absence data, Bray–Curtis distance, which take into account the abundances of species (here parasites) across different sites (here host species) and both weighted and unweighted UniFrac distances.

Next, we tested for differences in geographic range size and host specificity among haemosporidian lineages that were either exclusive to local breeding birds, exclusive to passing migrants or were shared between the 2 groups. To quantify geographic range size, we used the *geosphere* package in R (Hijmans, 2022) to calculate the area of a polygon defined by the geographic coordinates of the sampling locations in North America for each lineage that we detected using the ‘areaPolygon’ function. Coordinates were extracted from the MalAvi database (Bensch *et al*., 2009). To test for differences in host specificity, we used a phylogenetic approach with the ‘ses.mpd’ function in the *picante* R package (Kembel *et al*., 2010) following the parameters described in Galen *et al*. (2022). The input for this analysis was a host–parasite interaction matrix (parasites as rows, hosts as columns) constructed from host–parasite associations obtained from the ‘hosts and sites’ table in the MalAvi database (accessed 26 July 2023) that was filtered to include only haemosporidian *cytb* lineages that we sampled. We used phylogenetic analysis of variance (ANOVA) to test for trait differences among haemosporidians that belonged to different host migratory categories using the ‘phylANOVA’ function from the *phytools* package (Revell, 2012).

Lastly, we tested whether haemosporidian lineages that were carried by migratory birds and are transmitted in North America outside of Rushton were associated with a significantly different climate than haemosporidians that we confirmed were transmitted at Rushton. Out of the 52 lineages that we detected at Rushton with confirmed North America transmission, 16 were found to be transmitted outside of Rushton (but not at Rushton), 23 were found to be transmitted at Rushton and 13 lineages did not have additional sampling sites in North America and so were not included in this analysis. We again queried records of the target haemosporidians from the ‘hosts and sites’ table in MalAvi for which sampling coordinates were available. We downloaded temperature variables from the MERRAclim dataset at a 2.5 arc-minute scale (Vega *et al*., 2017), and then extracted the temperature variables at each locality in R by creating a combined list of all variables using the *velox* package (version 0.2.1, Hunziker, 2021) and extracting the temperature variables by converting all coordinates to spatial points in *sp* (version 1.5-0, Pebesma and Bivand, 2005; Bivand *et al*., 2013) and using the ‘extract_points’ function in *sp*. We specifically focused on MERRAclim variable BIO10, which corresponds to the mean temperature of the warmest quarter, rather than all bioclimatic variables collectively because we had an *a priori* expectation from previous research that temperature is particularly influential for haemosporidian sporogonic development in the vector (Valkiūnas, 2004), and can potentially negatively impact transmission (Platonova and Palinauskas, 2021). We used phylogenetic ANOVA as above to test whether haemosporidians found in migratory birds and not transmitted at Rushton are associated with significantly lower BIO10 values than haemosporidians known to be transmitted at Rushton.

### Temporal associations among bird and vector abundances

To characterize the potential for vectors to transmit haemosporidians during migratory stopover periods at Rushton, we used publicly available databases of bird and mosquito abundances in southeastern Pennsylvania. Note that because no publicly available database of abundance values exists for vectors in the families Simuliidae and Ceratopogonidae, the vectors of *Leucocytozoon* and *Haemoproteus*, respectively, this analysis only concerns temporal overlap between migratory birds and the mosquito (Culicidae) vectors of *Plasmodium*.

We used the *ebirdst* R package (Strimas-Mackey *et al*., 2023) to generate estimated counts of migratory bird species that we sampled at Rushton across each week of the year. The counts estimated by *ebirdst* represent the expected counts of a bird species on a 1 h, 1 km checklist submitted to eBird during an optimal observation period, and are estimated for 2022 (Fink *et al*., 2023; Strimas-Mackey *et al*., 2023). We also used a publicly available database of mosquito counts from West Nile virus surveys across the state of Pennsylvania (Pennsylvania Department of Environmental Protection, accessed through https://files.dep.state.pa.us/Water/WNV/MosquitoTestingData/ in October 2023). This database includes exact counts of every species of mosquito that was trapped between the months of April and October across 14 901 sampling sites in Pennsylvania between 2016 and 2022. We restricted this dataset to only include: (1) mosquito counts from traps set in Chester County, Pennsylvania (the county in which Rushton is located), (2) mosquitoes sampled from gravid traps that collect mosquitoes that have putatively already fed on a host, which reflects the proportion of a mosquito population that is potentially involved in disease transmission and (3) mosquito species that have been confirmed as vectors of avian malaria parasites based on the review by Santiago-Alarcon *et al*. (2012). We summarized cumulative counts of mosquito species for each week of the sampling season, and corrected for differential sampling efforts across weeks by randomly extracting the smallest number of independent sampling events that occurred during any week of the year (95 sampling events), without replacement. We repeated this subsampling protocol 1000 times and used the average number of mosquitos counted per week from the subsampling procedure for analysis.

Using the estimated weekly counts for migratory bird species and mosquitoes for weeks 18 through 39 of the annual calendar (corresponding approximately to the last week of April through the last week of September), we used cross-correlation analysis to test whether periods of migratory bird and mosquito abundance are decoupled over time. Rather than assess the abundance of all migratory bird species at Rushton, we focused on migratory species that we sampled in the 3 avian families that made up the overwhelming majority (93%) of migrant individuals that were sampled at Rushton: Parulidae, Passerellidae and Turdidae. We used the ‘cc.test’ function from the *testcorr* package (Dalla *et al*., 2021) to test for the correlation between migratory bird and mosquito abundances over time.

## Results

### Haemosporidian abundance and diversity

**Figure 1.**
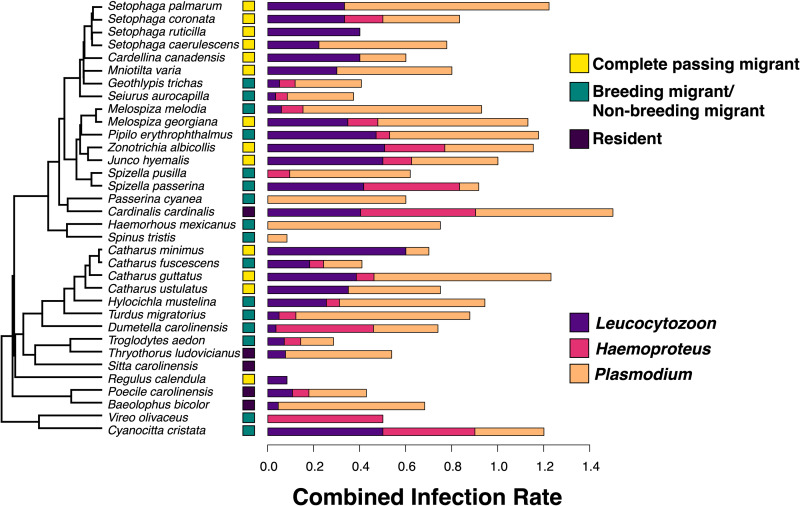
Haemosporidian prevalence *of Leucocytozoon*, *Haemoproteus* and *Plasmodium* in bird species that were sampled at Rushton at least 5 times. Each bird species is classified as a resident (does not exhibit migratory behaviour), a breeding migrant/non-breeding migrant (migratory species that both breed at Rushton and pass through as migrants) or a complete passing migrant (are migratory and are not known to breed at Rushton). The combined haemosporidian infection rate is depicted, which equals the average number of haemosporidian infections per individual of a host species. The combined infection rate is shown as the additive prevalences of *Leucocytozoon*, *Haemoproteus* and *Plasmodium* within each host species.

We sampled 1454 birds of 63 species between 2015 and 2019. We restricted this dataset to the 1404 samples of birds in the order Passeriformes due to low sample sizes of non-passerine hosts. Haemosporidian prevalence in individual host species varied widely (Fig. 1). Among residents, prevalence varied from 0% (*Sitta carolinensis*) to over 90% (*Cardinalis cardinalis*). We observed similar ranges of prevalence in migratory host species: breeding migrants at Rushton varied from 8% prevalence (*Spinus tristis*) to 90% (*Cyanocitta cristata*), whereas complete passing migrants varied from 8% (*Regulus calendula*) to 100% (*Setophaga palmarum*). Across all samples, the overall haemosporidian prevalence was 63.1%. *Plasmodium* was the most commonly encountered parasite with a prevalence of 38.6%, followed by *Haemoproteus* (including *Parahaemoproteus*) at 22.4% and *Leucocytozoon* at 16.8%. Coinfections were common, with 21% of all hosts harbouring multiple haemosporidian infections.

We recorded 91 haemosporidian *cytb* lineages (20 *Haemoproteus*, 41 *Leucocytozoon*, 30 *Plasmodium*). Out of 1276 individual haemosporidian detections (including coinfections), 54% were of just 5 common lineages: MAFUS02 (*Haemoproteus*; 204 detections), PADOM11 (*Plasmodium*; 185 detections), TUMIG03 (*Plasmodium*; 111 detections), SEIAUR01 (*Plasmodium*; 102 detections) and GEOTRI09 (*Plasmodium*; 93 detections). Twenty-seven lineages were novel, though these lineages were typically rare (5 or fewer detections) and were mostly different from a more common lineage by 1 base pair (22 of the 27 lineages). All lineage infection data have been deposited in the MalAvi database (Bensch *et al*., 2009), and the sequences for novel lineages have been deposited in GenBank (accession numbers OQ503649–OQ503676). After lumping together similar lineages for analysis, we retained 63 lineages (18 *Haemoproteus*, 25 *Leucocytozoon*, 20 *Plasmodium*). We confirmed North American transmission of 52 of these lineages, 26 of which were confirmed as transmitted at Rushton (Supplementary Table 1). We confirmed that the remaining parasite lineages were transmitted in North America outside of Rushton through our detection of parasites in migrating hatch-year birds during autumn (meaning that they had acquired their infections before ever leaving North America), or from previous records deposited in MalAvi of infections of these lineages in non-migratory bird species in North America (Supplementary Table 1).

### Haemosporidian community change across migratory host groups

We classified 557 host individuals as local breeding birds at Rushton (130 residents and 427 breeding migrants), and 737 individuals were classified as passing migrants that do not breed at Rushton and instead use Rushton as a stopover site (283 were complete passing migrants, and 454 were non-breeding migrants). We were unable to classify 110 birds as either breeding migrants or non-breeding migrants, as they were sampled at Rushton during a time when some members of the species are breeding locally and some are still passing through as migrants.

**Figure 2.**
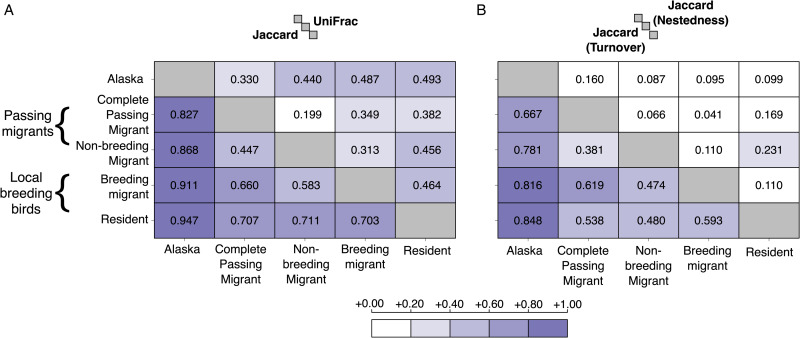
Haemosporidian beta diversity across avian migratory categories at Rushton. ‘Alaska’ includes the haemosporidian community found in breeding birds in Alaska by Galen *et al*. (2019), ‘Complete passing migrant’ is the haemosporidian community sampled in hosts at Rushton that are migratory and do not breed at Rushton, ‘Non-breeding migrant’ includes haemosporidians sampled in passing migrants of species that do breed at Rushton, ‘Breeding migrant’ is the haemosporidian community sampled in migratory host species that were breeding at Rushton and ‘Resident’ is the haemosporidian community of local breeding birds at Rushton that are non-migratory. (A) Lower triangle is the Jaccard distance and the upper triangle is the unweighted UniFrac distance. (B) Lower triangle is the turnover component of the Jaccard distance depicted in panel A and the upper triangle is the nestedness component of the Jaccard distance depicted in panel A.

Haemosporidian communities were distinct between local breeding birds and passing migrants at Rushton. Jaccard dissimilarity between the 2 groups was driven primarily by turnover, not nestedness (total beta diversity = 0.58, nestedness = 0.09, turnover = 0.49). When breaking down bird species into 4 migratory categories (residents, breeding migrants, non-breeding migrants and complete passing migrants), high Jaccard dissimilarity was seen between resident species and all other migratory categories (Fig. 2A). Again, beta diversity among the 4 migratory categories was driven primarily by turnover, not nestedness (Fig. 2B). Notably, the non-breeding migrant haemosporidian community was more similar to that of complete passing migrants than it was to breeding migrants, despite the fact that non-breeding migrants and breeding migrants contain overlapping host species. We also compared the haemosporidian community of passing migrants and local breeding birds at Rushton to a high-latitude haemosporidian community that was previously sampled in Alaska. Using the Jaccard index (Fig. 2) and unweighted UniFrac distances, we found that the haemosporidians of passing migrants were more similar to haemosporidians sampled in Alaska than they were to haemosporidians sampled in breeding birds at Rushton (Fig. 2). PERMANOVA indicated that the haemosporidian communities in local breeding birds and passing migratory birds were significantly different, no matter the index used to characterize beta diversity (Jaccard index: *F* = 1.99, *P* = 0.001; Bray–Curtis index: *F* = 2.54, *P* = 0.002; unweighted UniFrac: *F* = 3.03, *P* = 0.012; weighted UniFrac: *F* = 4.08, 0.011).

There was no difference in the geographic distribution of haemosporidians that infected local breeding birds only, passing migrants only or were shared between the 2 groups (phylogenetic ANOVA: *F* = 2.0, *P* = 0.22). We also found no difference in host specificity among haemosporidians from different avian migratory categories (phylogenetic ANOVA: *F* = 2.3, *P* = 0.14).

**Figure 3.**
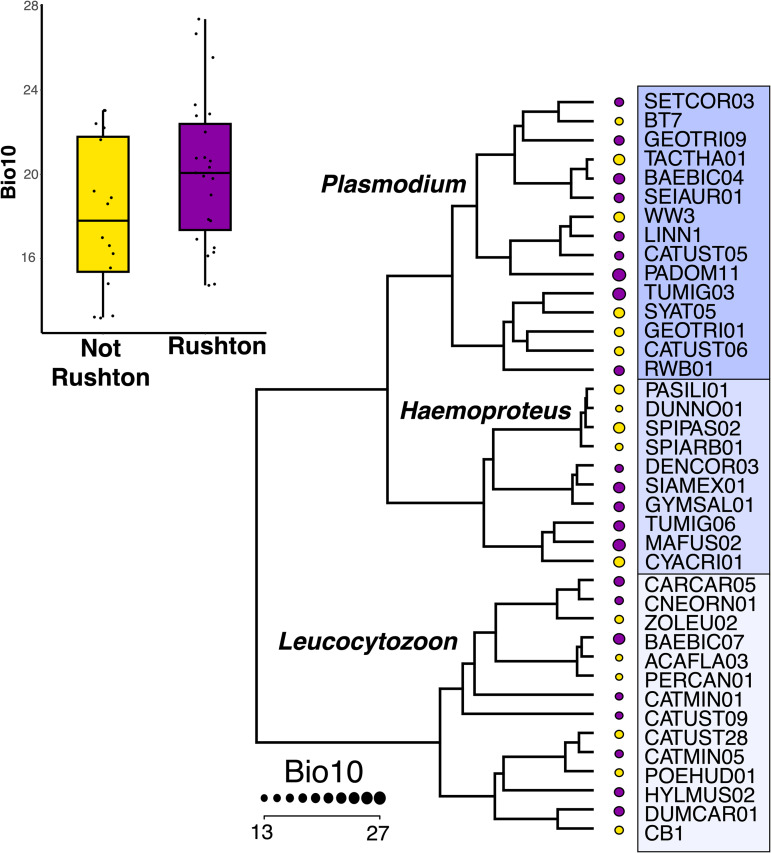
Haemosporidian lineages detected in passing migrants that are transmitted in North America, but not at Rushton (‘Not Rushton’, shown in yellow), are associated with sampling sites with lower mean temperatures of the warmest quarter (MERRAclim Bio10) than haemosporidians that we confirmed are transmitted at Rushton (‘Rushton’, shown in purple). Mean MERRAclim Bio10 values (which corresponds to the mean temperature of the warmest quarter) are depicted next to each haemosporidian lineages in the haemosporidian phylogeny, and as a boxplot.

Haemosporidians that we confirmed were transmitted at Rushton were associated with additional sampling sites that have significantly higher mean temperatures of the warmest quarter than haemosporidians found in passing migrants that are transmitted in North America outside of Rushton (Fig. 3; phylogenetic ANOVA: *F* = 3.18, *P* = 0.047).

### Temporal overlap between migratory bird and mosquito abundance

**Figure 4.**
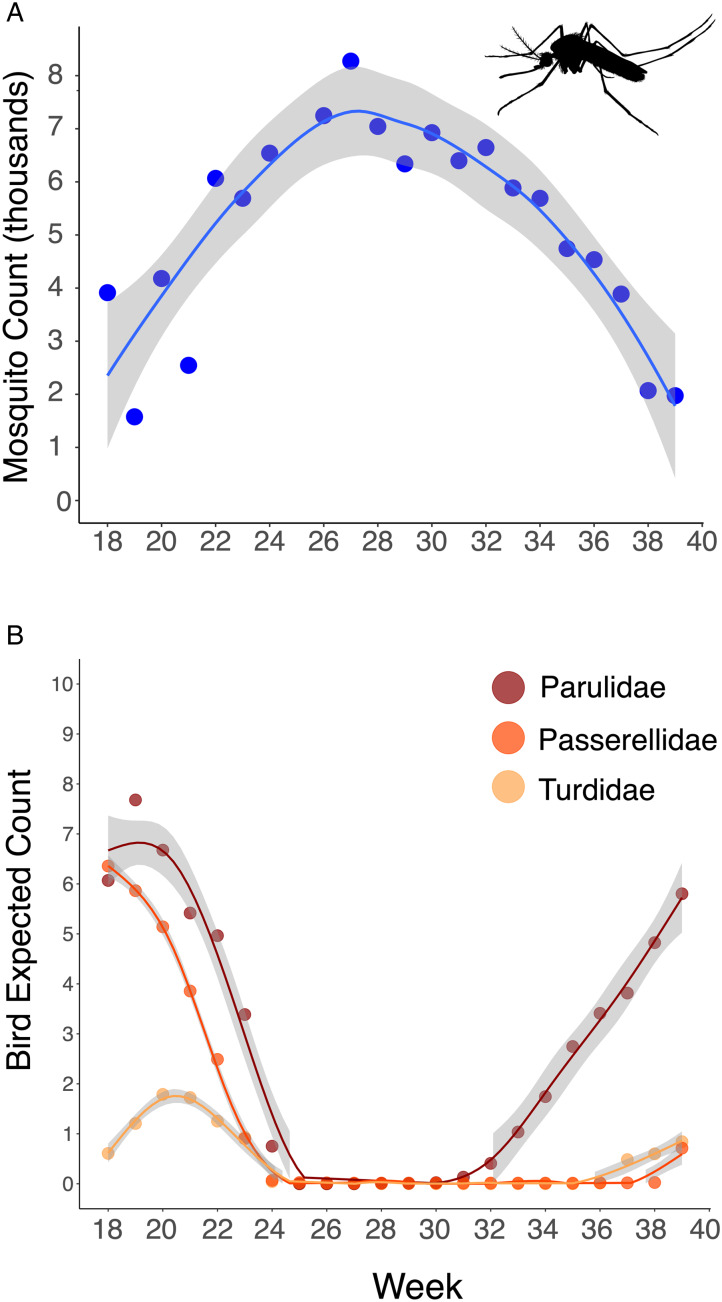
Mosquito and migratory bird abundance over time in southeastern Pennsylvania. (A) Cumulative mosquito count per week based on random sampling of 95 trapping events for annual weeks 18 through 39 between 2016 and 2022. (B) Expected counts of migratory birds in the families Parulidae, Passerellidae and Turdidae at Rushton during weeks 18 through 39 of the annual calendar for the year 2022. Mosquito silhouette was downloaded from www.phylopic.org.

The abundance of all 3 common migratory bird families (Turdidae, Parulidae and Passerellidae) was significantly negatively correlated with mosquito abundance at a time lag of zero, indicating that when bird abundance was high, mosquito abundance was low and vice versa for each week of the study period (Turdidae: *t* = −2.9, *P* = 0.003; Parulidae: *t* = −4.1, *P* ≤ 0.001; Passerellidae: *t* = −2.5, 0.014) (Fig. 4). For all 3 bird families, significant positive correlations with mosquito abundance only occurred at time lags of 5 weeks or more, showing a significant temporal offset between when migratory bird abundance and mosquito abundance peak. We repeated this analysis using all mosquito species, including those that have been confirmed as vectors of avian malaria parasites and those that have not, and found virtually identical results (Supplementary Fig. 1). When considering mosquito species individually, we found that a single mosquito species, *Culex restuans*, exhibited positive correlations with avian abundance (Supplementary Fig. 2, Table 2). *Culex restuans* was significantly positively correlated with the avian family Turdidae at a time lag of zero (*t* = 2.4, *P* = 0.017), though correlations with the families Parulidae and Passerellidae were not significant and all other mosquito species exhibited only negative correlations.

## Discussion

Migratory animals clearly influence the global distributions of parasites and are often studied for their potential to spread their parasites throughout their annual cycle (Pulgarín-R *et al*., 2019; Soares *et al*., 2020; Poulin and de Angeli Dutra, 2021). Of particular interest is the capacity for migratory animals to spread parasites to local hosts during active migration at stopover sites, yet this phenomenon has rarely been investigated in most host–parasite systems. We studied haemosporidians infecting birds that stop briefly at a migratory stopover site to determine the potential for the spread of these parasites between passing migrants and the local bird community. We found multiple lines of evidence to suggest that there is limited potential for transmission of haemosporidians between birds that breed at a temperate forest site and birds that use this site as a temporary stopover location.

Our first goal was to determine whether birds breeding at Rushton and birds using this site for stopover have similar haemosporidian parasite communities. We found that local breeding birds and passing migrants do share a core community of abundant and widespread haemosporidians, with 5 haemosporidian lineages that were shared between local breeding and migrant birds composing over half of all haemosporidian detections: *Haemoproteus* MAFUS02 and *Plasmodium* PADOM11, SEIAUR01, TUMIG03 and GEOTRI09. All 5 of these lineages have been recorded dozens of times from multiple sites in North America, and we confirmed that they are transmitted at Rushton. The existence of this group of abundant, widespread haemosporidians suggests that there is potential for the transmission of these lineages between local and passing migrant birds at Rushton. Although transmission of these abundant haemosporidians during migratory stopover is not expected to lead to disease emergence in communities where these lineages already occur, there is still risk of the introduction of these abundant parasites to novel communities by migratory birds, particularly due to vagrancy (Cohen *et al*., 2015).

However, when considering all haemosporidian lineages, multiple beta diversity metrics indicated that the haemosporidian communities of local breeding birds at Rushton and passing migrants are significantly different, whether considering presence/absence, abundances or phylogenetic relationships. The existence of a distinct haemosporidian community in passing migrants at our temperate sampling site was expected, given that many of these host species are known to breed in the North American boreal forest. Recent surveys of haemosporidians in North American boreal forest communities have identified high haemosporidian diversity that is largely distinct from temperate sampling sites (Oakgrove *et al*., 2014; Galen *et al*., 2019; Fecchio *et al*., 2020). Indeed, by comparing the haemosporidian community of passing migrants at Rushton to a haemosporidian community from Alaska, we found evidence that passing migrants sampled at Rushton harbour haemosporidians that are characteristic of high-latitude boreal forests in North America. Collectively, these patterns support the inference that haemosporidians, like their avian hosts, are biogeographically structured in North America as has been shown in South America (Fecchio *et al*., 2019; McNew *et al*., 2021).

What factors have allowed migrant birds at Rushton to maintain a distinct haemosporidian community, despite the fact that these birds clearly intermingle with temperate breeding birds during stopover? Our study suggests that there are several important factors that likely limit the transmission of haemosporidians between migratory and local birds at Rushton and other similar temperate stopover sites. First, there appears to be either a direct or an indirect effect of climate on haemosporidian distributions that may limit transmission potential at stopover sites. Haemosporidians found in passing migrants, for which we do not have evidence of transmission at Rushton, were associated with a distinct climate relative to haemosporidians that are transmitted at Rushton. Specifically, the haemosporidians found in passing migrants were generally recorded at sites with lower temperatures during the warmest quarter of the year. In fact, based on current sampling records (e.g. *Haemoproteus* SPIARB01 and DUNNO01; *Leucocytozoon* ACAFLA03, PERCAN01 and POEHUD01; *Plasmodium* BT7), several haemosporidians that we only found in passing migrants appear to have distributions that are restricted to the boreal zone of North America, suggesting the existence of some barrier to transmission south of this region. One variable that likely structures the distributions of North American haemosporidians is the distribution of their vectors. Haemosporidians not only exhibit specificity at the level of the vector family, but some haemosporidians appear to be transmitted by specific vector genera or clades within a vector genus (Santiago-Alarcon *et al*., 2012). The lack of overlap in vector communities between boreal and temperate zones in North America is likely an important contributor to the geographic structure of haemosporidian communities, and may act as a limitation to transmission potential during avian stopover.

In addition to the species composition of the available vector community, a related factor that likely limits the potential for haemosporidian transmission during stopover is vector abundance. For the avian families Turdidae, Parulidae and Passerellidae, which make up the majority of migratory birds that stop temporarily at Rushton, migrant abundance is negatively correlated with overall mosquito abundance throughout the year. For all 3 host families, counts of migratory species peak from late April into mid-May, when mosquito abundance is still low (though we note that the abundance of 1 mosquito species, *C. restuans*, appears to be an exception to this general pattern). By the time mosquito abundance takes off in early June, migratory birds are virtually gone from the study region. Similarly, mosquito abundance in southeastern Pennsylvania declines sharply in mid-September, just as the abundance of the families Turdidae and Passerellidae begin to climb and the abundance of Parulidae is still increasing. Although these patterns do not preclude transmission from occurring, it does suggest that the probability of a successful transmission event between a migratory and local host at Rushton is negatively impacted by mosquito abundance given that it has been documented that *Plasmodium* transmission increases with vector density (Koella, 1991). Furthermore, the brief periods in the spring and autumn when migratory birds are abundant at Rushton may not be ideal for the development of some haemosporidians in the vector. For instance, temperatures below 15°C, which occur regularly at Rushton particularly during the late spring, appear to significantly delay sporozoite development in some *Plasmodium* and *Haemoproteus* species and also inhibit vector biting activity (Atkinson *et al*., 1988; LaPointe *et al*., 2010). Unfortunately, we were unable to analyse vector abundance for the simuliid blackflies and *Culicoides* biting midges, which are the vectors for *Leucocytozoon* and *Haemoproteus*, respectively. Although this does limit our inferences regarding transmission potential for *Plasmodium*, previous surveys in eastern North America suggest that simuliid and *Culicoides* abundances could also be decoupled from migratory bird abundance at temperate North American sites (simuliids: Adler *et al*., 1982; *Culicoides*: Lysyk, 2006).

There are numerous intriguing avenues for future research into the potential for migratory animals to spread pathogens at stopover locations. One natural extension of this research is to evaluate the temporal overlap between migratory bird and vector abundances across latitudes, as one might hypothesize that the window of migrant-vector overlap is extended at lower latitudes in North America. For example, in subtropical regions of North America, the abundance of some vectors may be positively correlated with migratory bird abundance as the vectors reach seasonal peaks during the brief window in which most migratory birds pass through. Modelling the effect of climate change on the interaction between vectors and migratory hosts has shown that these windows of time during which migrants and vectors can interact may change over time (Hall *et al*., 2016), and so the effects of warming temperatures should be incorporated in future studies. Lastly, from an evolutionary perspective, it is intriguing to consider whether migratory birds in North America have optimized the timing of their migratory pathways to reduce the temporal overlap with vectors. We were also unable to factor the length of time that migrant birds linger at stopover sites into this analysis. It is interesting to consider that if transmission of vector-mediated pathogens occurs at stopover sites, it may be more likely to occur in the autumn due to the influx of naïve hatch-year birds and the tendency for birds migrating in the autumn to engage in longer stopover durations (Nilsson *et al*., 2013). Collectively, our understanding of pathogen transmission during migratory stopover is still in its infancy and is deserving of increased focus.

## Supporting information

Galen et al. supplementary material 1 Galen et al. supplementary material

Galen et al. supplementary material 2 Galen et al. supplementary material

## Data Availability

Genetic data for novel haemosporidian lineages generated by this study have been made available on GenBank (accession numbers: OQ503649–OQ503676). Data for all haemosporidian detections have been submitted to the MalAvi database. The data file and R code used for analysis have been included as Supplementary materials. The accession numbers for the blood smears that were produced for this research are included in the Supplementary data file, and the original blood smears are available for study at the Academy of Natural Sciences of Drexel University.

## Supplementary material

The supplementary material for this article can be found at https://doi.org/10.1017/S0031182024001239.

## References

[ref1] Adler PH, Travis BL, Kim KC and Masteller EC (1982) Seasonal emergence patterns of black flies (Diptera: Simuliidae) in northwestern Pennsylvania. The Great Lakes Entomologist 15, 253–260. doi: 10.22543/0090-0222.1448

[ref2] Altizer S, Bartel R and Han BA (2011) Animal migration and infectious disease risk. Science (New York, N.Y.) 331, 296–302. 21252339 10.1126/science.1194694

[ref3] Atkinson C, Forrester D and Greiner E (1988) Epizootiology of *Haemoproteus meleagridis* (Protozoa: Haemosporina) in Florida: Seasonal transmission and vector abundance. Journal of Medical Entomology 25, 45–51. 3128662 10.1093/jmedent/25.1.45

[ref4] Balstad LJ, Binning SA, Craft ME, Zuk M and Shaw AK (2021) Parasite intensity and the evolution of migratory behavior. Ecology 102, e03229. 33098657 10.1002/ecy.3229

[ref5] Baselga A and Orme CDL (2012) Betapart: an R package for the study of beta diversity. Methods in Ecology and Evolution 3, 808–812.

[ref6] Bensch S, Pérez-Tris J, Waldenström J and Hellgren O (2004) Linkage between nuclear and mitochondrial DNA sequences in avian malaria parasites: multiple cases of cryptic speciation? Evolution 58, 1617–1621. 15341164 10.1111/j.0014-3820.2004.tb01742.x

[ref7] Bensch S, Hellgren O and Pérez-Tris J (2009) MalAvi: a public database of malaria parasites and related haemosporidians in avian hosts based on mitochondrial cytochrome b lineages. Molecular Ecology Resources 9, 1353–1358. 21564906 10.1111/j.1755-0998.2009.02692.x

[ref8] Bivand RS, Pebesma E and Gómez-Rubio V (2013) Applied Spatial Data Analysis with R. New York, NY: Springer, doi: 10.1007/978-1-4614-7618-4

[ref9] Bouckaert R, Heled J, Kühnert D, Vaughan T, Wu C-H, Xie D, Suchard MA, Rambaut A and Drummond AJ (2014) BEAST 2: a software platform for Bayesian evolutionary analysis. PLoS Computational Biology 10, e1003537. 24722319 10.1371/journal.pcbi.1003537 PMC3985171

[ref10] Boulinier T, Kada S, Ponchon A, Dupraz M, Dietrich M, Gamble A, Bourret V, Duriez O, Bazire R, Tornos J, Tveraa T, Chambert T, Garnier R and McCoy KD (2016) Migration, prospecting, dispersal? What host movement matters for infectious agent circulation? Integrative and Comparative Biology 56, 330–342. 27252195 10.1093/icb/icw015

[ref11] Bradley CA and Altizer S (2005) Parasites hinder monarch butterfly flight: implications for disease spread in migratory hosts. Ecology Letters 8, 290–300.

[ref12] Briscoe AG, Nichols S, Hartikainen H, Knipe H, Foster R, Green AJ, Okamura B and Bass D (2022) High-throughput sequencing of faeces provides evidence for dispersal of parasites and pathogens by migratory waterbirds. Molecular Ecology Resources 22, 1303–1318. 34758191 10.1111/1755-0998.13548

[ref13] Carlson ML, Proudfoot GA, Gentile K, Dispoto J and Weckstein JD (2018) Haemosporidian prevalence in northern saw-whet owls *Aegolius acadicus* is predicted by host age and average annual temperature at breeding grounds. Journal of Avian Biology 49, e01817.

[ref14] Ciloglu A, Ergen AG, Inci A, Dik B, Duzlu O, Onder Z, Yetismis G, Bensch S, Valkiūnas G and Yildirim A (2020) Prevalence and genetic diversity of avian haemosporidian parasites at an intersection point of bird migration routes: Sultan Marshes National Park, Turkey. Acta Tropica 210, 105465. 32504592 10.1016/j.actatropica.2020.105465

[ref15] Cohen EB, Auckland LD, Marra PP and Hamer SA (2015) Avian migrants facilitate invasions of neotropical ticks and tick-borne pathogens into the United States. Applied and Environmental Microbiology 81. doi: 10.1128/AEM.02656-15 PMC4644638 26431964

[ref16] Dalla V and Phillips LG and PCB (2021) testcorr: Testing zero correlation. R package version 0.2.0, https://CRAN.R-project.org/package=testcorr.

[ref17] Darriba D, Taboada GL, Doallo R and Posada D (2012) Jmodeltest 2: more models, new heuristics and high-performance computing. Nature Methods 9, 772. 10.1038/nmeth.2109 PMC4594756 22847109

[ref18] de Angeli Dutra D, Filion A, Fecchio A, Braga ÉM and Poulin R (2021a) Migrant birds disperse haemosporidian parasites and affect their transmission in avian communities. Oikos 130, 979–988.

[ref19] de Angeli Dutra D, Fecchio A, Martins Braga É and Poulin R (2021b) Migratory birds have higher prevalence and richness of avian haemosporidian parasites than residents. International Journal for Parasitology 51, 877–882. 33848498 10.1016/j.ijpara.2021.03.001

[ref20] Emmenegger T, Bauer S, Dimitrov D, Olano Marin J, Zehtindjiev P and Hahn S (2018) Host migration strategy and blood parasite infections of three sparrow species sympatrically breeding in Southeast Europe. Parasitology Research 117, 3733–3741. 30232606 10.1007/s00436-018-6072-7

[ref21] Fecchio A, Bell JA, Pinheiro RBP, Cueto VR, Gorosito CA, Lutz HL, Gaiotti MG, Paiva LV, França LF, Toledo-Lima G, Tolentino M, Pinho JB, Tkach VV, Fontana CS, Grande JM, Santillán MA, Caparroz R, Roos AL, Bessa R, Nogueira W, Moura T, Nolasco EC, Comiche KJM, Kirchgatter K, Guimarães LO, Dispoto JH, Marini MÂ, Weckstein JD, Batalha-Filho H and Collins MD (2019) Avian host composition, local speciation and dispersal drive the regional assembly of avian malaria parasites in South American birds. Molecular Ecology 28, 2681–2693. 30959568 10.1111/mec.15094

[ref22] Fecchio A, Bell JA, Bosholn M, Vaughan JA, Tkach VV, Lutz HL, Cueto VR, Gorosito CA, González-Acuña D, Stromlund C, Kvasager D, Comiche KJM, Kirchgatter K, Pinho JB, Berv J, Anciães M, Fontana CS, Zyskowski K, Sampaio S, Dispoto JH, Galen SC, Weckstein JD and Clark NJ (2020) An inverse latitudinal gradient in infection probability and phylogenetic diversity for *Leucocytozoon* blood parasites in New World birds. Journal of Animal Ecology 89, 423–435. 31571223 10.1111/1365-2656.13117

[ref23] Fecchio A, Clark NJ, Bell JA, Skeen HR, Lutz HL, De La Torre GM, Vaughan JA, Tkach VV, Schunck F, Ferreira FC, Braga ÉM, Lugarini C, Wamiti W, Dispoto JH, Galen SC, Kirchgatter K, Sagario MC, Cueto VR, González-Acuña D, Inumaru M, Sato Y, Schumm YR, Quillfeldt P, Pellegrino I, Dharmarajan G, Gupta P, Robin VV, Ciloglu A, Yildirim A, Huang X, Chapa-Vargas L, Álvarez-Mendizábal P, Santiago-Alarcon D, Drovetski SZ, Hellgren O, Voelker G, Ricklefs RE, Hackett SJ, Collins MD, Weckstein JD and Wells K (2021) Global drivers of avian haemosporidian infections vary across zoogeographical regions. Global Ecology and Biogeography 30, 2393–2406.

[ref24] Figuerola J and Green AJ (2000) Haematozoan parasites and migratory behaviour in waterfowl. Evolutionary Ecology 14, 143–153.

[ref25] Fink D, Auer T, Johnston A, Strimas-Mackey M, Ligocki S, Robinson O, Hochachka W, Jaromczyk L, Crowley C, Dunham K, Stillman A, Davies I, Rodewald A, Ruiz-Gutierrez V and Wood C (2023) eBird Status and Trends, Data Version: 2022; Released: 2023. Ithaca, New York: Cornell Lab of Ornithology, doi:/10.2173/ebirdst.2022

[ref26] Fritzsche McKay A and Hoye BJ (2016) Are migratory animals superspreaders of infection? Integrative and Comparative Biology 56, 260–267. 27462034 10.1093/icb/icw054

[ref27] Galen SC, Nunes R, Sweet PR and Perkins SL (2018) Integrating coalescent species delimitation with analysis of host specificity reveals extensive cryptic diversity despite minimal mitochondrial divergence in the malaria parasite genus *Leucocytozoon*. BMC Evolutionary Biology 18, 128. 30165810 10.1186/s12862-018-1242-x PMC6117968

[ref28] Galen SC, Speer KA and Perkins SL (2019) Evolutionary lability of host associations promotes phylogenetic overdispersion of co-infecting blood parasites. Journal of Animal Ecology 88, 1936–1949. 31408525 10.1111/1365-2656.13089

[ref29] Galen SC, Ray S, Henry M and Weckstein JD (2022) Parasite-associated mortality in birds: the roles of specialist parasites and host evolutionary distance. Biology Letters 18, 20210575. 35414225 10.1098/rsbl.2021.0575 PMC9006019

[ref30] Hahn S, Bauer S, Dimitrov D, Emmenegger T, Ivanova K, Zehtindjiev P and Buttemer WA (2018) Low intensity blood parasite infections do not reduce the aerobic performance of migratory birds. Proceedings of the Royal Society B: Biological Sciences 285, 20172307. 10.1098/rspb.2017.2307 PMC5805937 29386365

[ref31] Hall RJ, Brown LM and Altizer S (2016) Modeling vector-borne disease risk in migratory animals under climate change. Integrative and Comparative Biology 56, 353–364. 27252225 10.1093/icb/icw049

[ref32] Hegemann A, Alcalde Abril P, Sjöberg S, Muheim R, Alerstam T, Nilsson J-Å and Hasselquist D (2018) A mimicked bacterial infection prolongs stopover duration in songbirds – but more pronounced in short- than long-distance migrants. Journal of Animal Ecology 87, 1698–1708. 30101481 10.1111/1365-2656.12895

[ref33] Hellgren O, Waldenström J, Peréz-Tris J, Szöll E, Si Ö, Hasselquist D, Krizanauskiene A, Ottosson U and Bensch S (2007) Detecting shifts of transmission areas in avian blood parasites – a phylogenetic approach. Molecular Ecology 16, 1281–1290. 17391413 10.1111/j.1365-294X.2007.03227.x

[ref34] Hellgren O, Atkinson CT, Bensch S, Albayrak T, Dimitrov D, Ewen JG, Kim KS, Lima MR, Martin L, Palinauskas V, Ricklefs R, Sehgal RNM, Valkiūnas G, Tsuda Y and Marzal A (2015) Global phylogeography of the avian malaria pathogen *Plasmodium relictum* based on MSP1 allelic diversity. Ecography 38, 842–850.

[ref35] Hijmans R (2022). geosphere: Spherical trigonometry. R package version 1.5-18, https://CRAN.R-project.org/package=geosphere.

[ref36] Hoberg EP and Brooks DR (2008) A macroevolutionary mosaic: episodic host-switching, geographical colonization and diversification in complex host–parasite systems. Journal of Biogeography 35, 1533–1550.

[ref37] Huang X, Chen Z, Guocheng Y, Xia C, Luo Q and Dong L (2022) Assemblages of *Plasmodium* and related parasites in birds with different migration statuses. International Journal of Molecular Sciences 23, 10277. 36142189 10.3390/ijms231810277 PMC9499606

[ref38] Hunziker P (2021) velox: Fast raster manipulation and extraction. R package version 0.2.1.

[ref39] Ishtiaq F and Renner SC (2020) Bird migration and vector-borne parasite transmission. In Santiago-Alarcon D and Marzal A (eds.), Avian Malaria and Related Parasites in the Tropics: Ecology, Evolution and Systematics. Cham: Springer International Publishing, pp. 513–526. doi: 10.1007/978-3-030-51633-8_16

[ref40] Jenkins T, Thomas GH, Hellgren O and Owens IPF (2012) Migratory behavior of birds affects their coevolutionary relationship with blood parasites. Evolution 66, 740–751. 22380437 10.1111/j.1558-5646.2011.01470.x

[ref41] Jetz W, Thomas GH, Joy JB, Hartmann K and Mooers AO (2012) The global diversity of birds in space and time. Nature 491, 444–448. 23123857 10.1038/nature11631

[ref42] Kembel SW, Cowan PD, Helmus MR, Cornwell WK, Morlon H, Ackerly DD, Blomberg SP and Webb CO (2010) Picante: R tools for integrating phylogenies and ecology. Bioinformatics (Oxford, England) 26, 1463–1464. 20395285 10.1093/bioinformatics/btq166

[ref43] Koella JC (1991) On the use of mathematical models of malaria transmission. Acta Tropica 49, 1–25. 1678572 10.1016/0001-706x(91)90026-g

[ref44] Krauss S, Stallknecht DE, Negovetich NJ, Niles LJ, Webby RJ and Webster RG (2010) Coincident ruddy turnstone migration and horseshoe crab spawning creates an ecological ‘hot spot’ for influenza viruses. Proceedings of the Royal Society B: Biological Sciences 277, 3373–3379. 10.1098/rspb.2010.1090 PMC2982236 20630885

[ref45] LaPointe DA, Goff ML and Atkinson CT (2010) Thermal constraints to the sporogonic development and altitudinal distribution of avian malaria *Plasmodium relictum* in Hawai'i. Journal of Parasitology 96, 318–324. 20001096 10.1645/GE-2290.1

[ref46] Loehle C (1995) Social barriers to pathogen transmission in wild animal populations. Ecology 76, 326–335.

[ref47] Lozupone C and Knight R (2005) UniFrac: a new phylogenetic method for comparing microbial communities. Applied and Environmental Microbiology 71, 8228–8235. 16332807 10.1128/AEM.71.12.8228-8235.2005 PMC1317376

[ref48] Lysyk TJ (2006) Abundance and species composition of *Culicoides* (Diptera: Ceratopogonidae) at cattle facilities in southern Alberta, Canada. Journal of Medical Entomology 43, 840–849. 17017217 10.1603/0022-2585(2006)43[840:aascoc]2.0.co;2

[ref49] Mages PA and Dill LM (2010) The effect of sea lice (*Lepeophtheirus salmonis*) on juvenile pink salmon (*Oncorhynchus gorbuscha*) swimming endurance. Canadian Journal of Fisheries and Aquatic Sciences 67, 2045–2051.

[ref50] McKinnon EA and Love OP (2018) Ten years tracking the migrations of small landbirds: lessons learned in the golden age of bio-logging. The Auk 135, 834–856.

[ref51] McMurdie PJ and Holmes S (2013) Phyloseq: an R package for reproducible interactive analysis and graphics of microbiome census data. PLoS ONE 8, e61217. 23630581 10.1371/journal.pone.0061217 PMC3632530

[ref52] McNew SM, Barrow LN, Williamson JL, Galen SC, Skeen HR, DuBay SG, Gaffney AM, Johnson AB, Bautista E, Ordoñez P, Schmitt CJ, Smiley A, Valqui T, Bates JM, Hackett SJ and Witt CC (2021) Contrasting drivers of diversity in hosts and parasites across the tropical Andes. Proceedings of the National Academy of Sciences 118, e2010714118. 10.1073/pnas.2010714118 PMC8000519 33731475

[ref53] Møller AP, de Lope F and Saino N (2004) Parasitism, immunity, and arrival date in a migratory bird, the Barn Swallow. Ecology 85, 206–219.

[ref54] Nilsson C, Klaassen RHG and Alerstam T (2013) Differences in speed and duration of bird migration between spring and autumn. The American Naturalist 181, 837–845. 10.1086/670335 23669545

[ref55] Oakgrove KS, Harrigan RJ, Loiseau C, Guers S, Seppi B and Sehgal RNM (2014) Distribution, diversity and drivers of blood-borne parasite co-infections in Alaskan bird populations. International Journal for Parasitology 44, 717–727. 25014331 10.1016/j.ijpara.2014.04.011

[ref56] Oksanen J, Simpson GL, Blanchet FG, Kindt R, Legendre P, Minchin P, O'Hara RB, Solymos P, Stevens MHH, Szoecs E, Wagner H, Barbour M, Bedward M, Bolker B, Borcard D, Carvalho G, Chirico M, Caceres MD, Durand S, Evangelista HBA, FitzJohn R, Friendly M, Furneaux B, Hannigan G, Hill MO, Lahti L, McGlinn D, Ouellette M-H, Cunha ER, Smith T, Stier A, Braak CJFT and Weedon J (2022) vegan: Community ecology package. R package version 2.6-4, https://CRAN.R-project.org/package=vegan

[ref57] Pebesma EJ and Bivand RS (2005) Classes and methods for spatial data in R. R News 5, 1–21.

[ref58] Platonova E and Palinauskas V (2021) The impact of temperature on the sporogonic development of the tropical avian malaria parasite *Plasmodium relictum* (genetic lineage pGRW4) in *Culex pipiens* form *molestus* mosquitoes. Microorganisms 9, 2240. 34835365 10.3390/microorganisms9112240 PMC8620208

[ref59] Poulin R and de Angeli Dutra D (2021) Animal migrations and parasitism: reciprocal effects within a unified framework. Biological Reviews 96, 1331–1348. 33663012 10.1111/brv.12704

[ref60] Pulgarín-R PC, Gómez C, Bayly NJ, Bensch S, FitzGerald AM, Starkloff N, Kirchman JJ, González-Prieto AM, Hobson KA, Ungvari-Martin J, Skeen H, Castaño MI and Cadena CD (2019) Migratory birds as vehicles for parasite dispersal? Infection by avian haemosporidians over the year and throughout the range of a long-distance migrant. Journal of Biogeography 46, 83–96.

[ref61] R Core Team (2022) R: A Language and Environment for Statistical Computing. Vienna, Austria: R Foundation for Statistical Computing, URL https://www.R-project.org/

[ref62] Revell LJ (2012) Phytools: an R package for phylogenetic comparative biology (and other things). Methods in Ecology and Evolution 3, 217–223.

[ref63] Santiago-Alarcon D, Palinauskas V and Schaefer HM (2012) Diptera vectors of avian haemosporidian parasites: untangling parasite life cycles and their taxonomy. Biological Reviews 87, 928–964. 22616880 10.1111/j.1469-185X.2012.00234.x

[ref64] Santiago-Alarcon D, Mettler R, Segelbacher G and Schaefer HM (2013) Haemosporidian parasitism in the blackcap *Sylvia atricapilla* in relation to spring arrival and body condition. Journal of Avian Biology 44, 521–530.

[ref65] Satterfield DA, Maerz JC, Hunter MD, Flockhart DTT, Hobson KA, Norris DR, Streit H, de Roode JC and Altizer S (2018) Migratory monarchs that encounter resident monarchs show life-history differences and higher rates of parasite infection. Ecology Letters 21, 1670–1680. 30152196 10.1111/ele.13144

[ref66] Soares L, Latta SC and Ricklefs RE (2020) Neotropical migratory and resident birds occurring in sympatry during winter have distinct haemosporidian assemblages. Journal of Biogeography 47, 748–759.

[ref67] Starkloff NC and Galen SC (2023) Coinfection rates of avian blood parasites increase with latitude in parapatric host species. Parasitology 150, 329–336. 36597832 10.1017/S0031182022001792 PMC10090641

[ref68] Strimas-Mackey M, Ligocki S, Auer T and Fink D (2023) ebirdst: Tools for loading, plotting, mapping and analysis of eBird status and trends data products. R package version 3.2022.0. Available at https://ebird.github.io/ebirdst/

[ref69] Sullivan BL, Wood CL, Iliff MJ, Bonney RE, Fink D and Kelling S (2009) Ebird: a citizen-based bird observation network in the biological sciences. Biological Conservation 142, 2282–2292.

[ref70] Valkiūnas G (2004) Avian Malaria Parasites and Other Haemosporidia. Boca Raton, FL: CRC Press.

[ref71] Vega GC, Pertierra LR and Olalla-Tárraga MÁ (2017) MERRAclim, a high-resolution global dataset of remotely sensed bioclimatic variables for ecological modelling. Scientific Data 4, 170078. 28632236 10.1038/sdata.2017.78 PMC5477563

