# Supplementary material for: Migratory birds have a distinct haemosporidian community and are temporally decoupled from vector abundance at a stopover site
Source: Parasitology. 2024 Nov 11;151(12):1326–35. doi: 10.1017/S0031182024001239 (PMC11893997; doi:10.1017/S0031182024001239)
Supplement: Galen et al. supplementary material 2 — Galen et al. supplementary material [file S0031182024001239sup002.docx]

**Migratory birds have a distinct haemosporidian community and are temporally decoupled from vector abundance at a stopover site**

**Supplementary Methods**

*Classification of hosts into migratory categories*

We classified four distinct categories of host migratory behavior among birds sampled at Rushton. First, we defined two categories within ‘local breeding birds’ that reproduce at Rushton: “residents” included birds that do not exhibit migratory behavior, and “breeding migrants” included birds that exhibit migratory behavior, but were confirmed to be breeding at Rushton. In contrast, “passing migrants” included “complete passing migrants” of bird species that are migratory and never reproduce at Rushton, and “non-breeding migrants” which included individuals of bird species that do reproduce at Rushton, but were passing through Rushton at the time of sampling. To distinguish “breeding migrants” (which reproduced at Rushton) from “non-breeding migrants” (which passed through Rushton), which can consist of the same bird species, we used date cutoffs, evidence of reproductive behavior, and evidence of site fidelity from banding records. We classified a migratory host as a “breeding migrant” if it exhibited at least one of the following traits: 1) it was sampled at Rushton between June 1 and August 10, 2) it exhibited a cloacal protuberance or brood patch, or 3) was sampled at Rushton multiple years in a row, indicating site fidelity. In contrast, we classified species that are known to breed at Rushton as “non-breeding migrants” if they were sampled between April 1 and May 10 or between September 1 and November 4 (the latest sampling date in our dataset) and did not exhibit evidence of reproductive behavior. Individuals from migratory species that breed at Rushton that were sampled between May 11 and May 31 or between August 11 and August 31 were considered to fall within an uncertain time period and were not classified unless they exhibited physical signs of reproductive behavior or site fidelity.


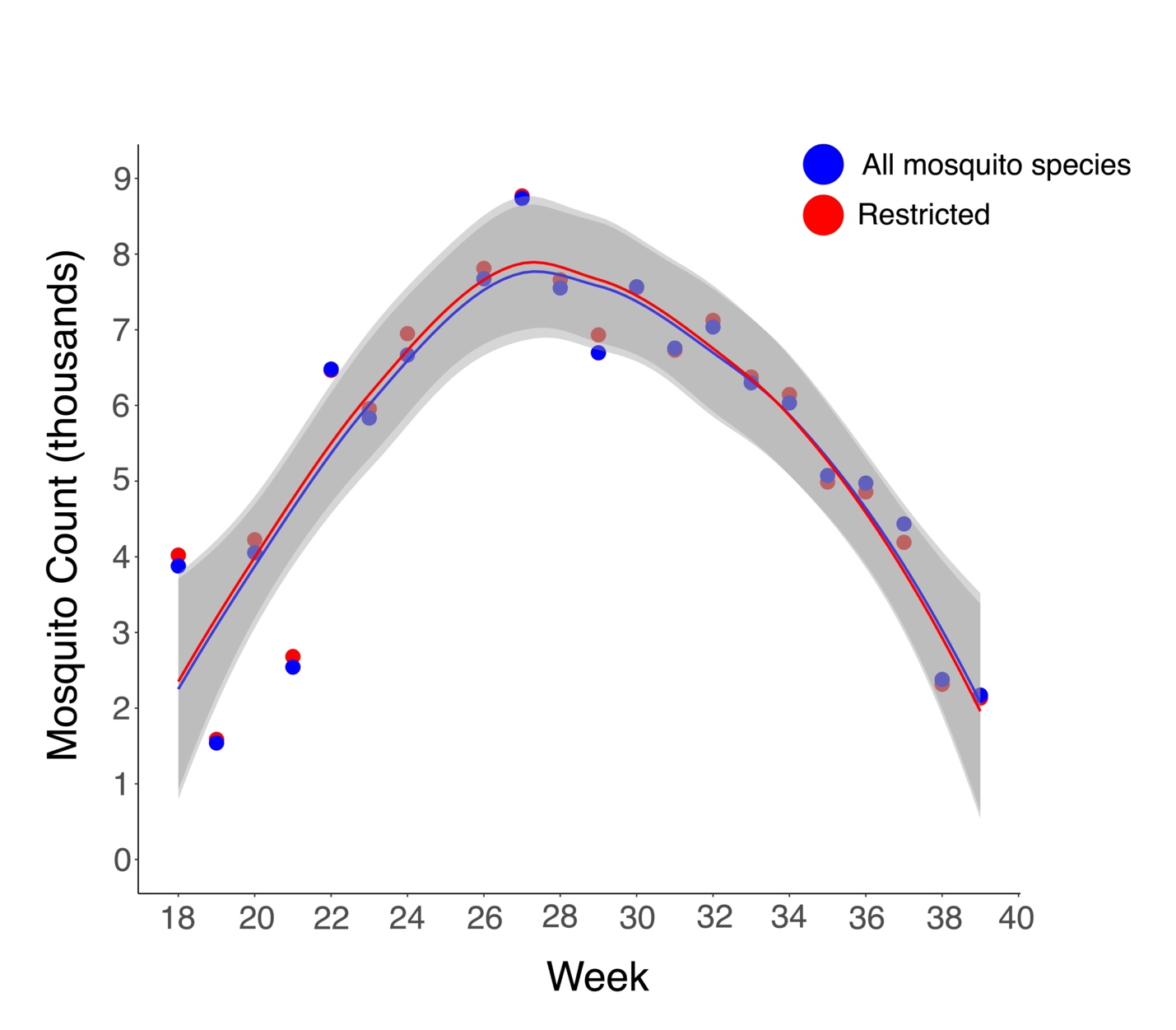


**Supplementary Figure 1**. A comparison of mosquito abundance over time in southeastern Pennsylvania using two sampling approaches. In blue are the cumulative counts of all mosquito species sampled based on the selection of 95 random trapping events per week for annual weeks 18 through 39 of the years 2016 to 2022. In red (“restricted”) are the cumulative counts for the same time period of only mosquito species that have been confirmed as vectors of avian malaria parasites based on Santiago-Alarcon et al. (2012).

**
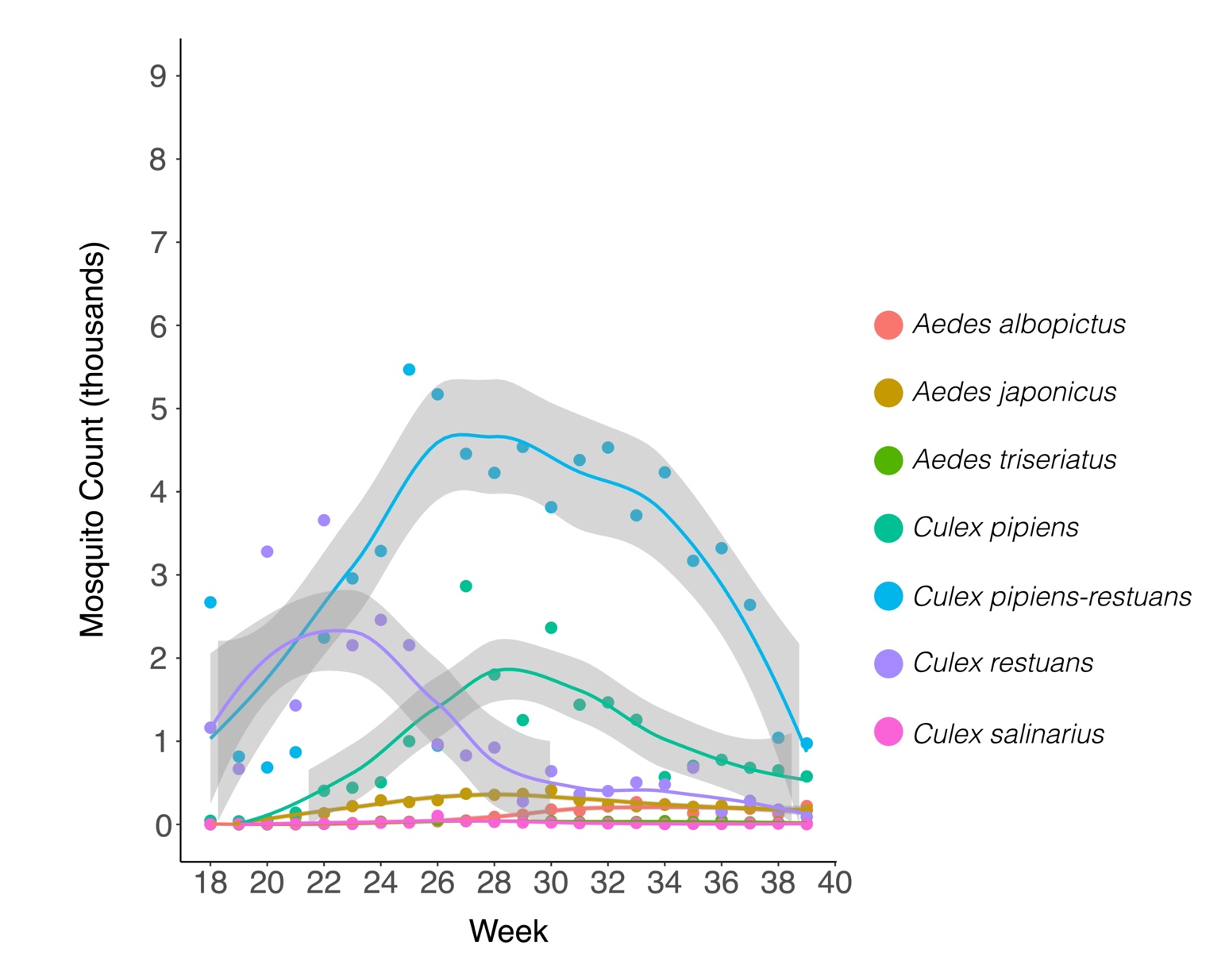
**

**Supplementary Figure 2**. A comparison of cumulative counts of seven mosquito species that were trapped at least 1,000 times between 2016 and 2022 in southeastern Pennsylvania. Cumulative counts are based on the selection of 95 random trapping events between week 18 and week 39 of the annual calendar from 2016 to 2022. “*Culex pipens-restuans*” represents mosquitos that could not be identified to either *Culex pipens* or *Culex restuans* due to their similar appearance.

**Supplementary Table 1.** Table of confirmed North American transmission for haemosporidian lineages sampled at Rushton. Shown is whether the lineage was confirmed to be transmitted at Rushton Woods Preserve, and the source of evidence that we used to confirm North American transmission.

| **Genus** | **Lineage** | **Rushton transmission** | **Evidence for North American transmission** |
| --- | --- | --- | --- |
| *Haemoproteus* | CARCAR12 | Yes | Found in resident species *Cardinalis cardinalis* |
| *Haemoproteus* | CYACRI01 |  | Found in *Cyanocitta cristata* which does not migrate outside of temperate North America |
| *Haemoproteus* | CYACRI02 |  | Found in *Cyanocitta cristata* which does not migrate outside of temperate North America |
| *Haemoproteus* | DENCOR03 | Yes | Found in resident species *Cardinalis cardinalis* |
| *Haemoproteus* | DUMCAR11 |  | Found in hatch year *Dumetella carolinensis* during fall migration |
| *Haemoproteus* | DUNNO01 |  | Found in hatch year birds of multiple species in the fall |
| *Haemoproteus* | GYMSAL01 | Yes | Found in resident species *Poecile carolinensis* |
| *Haemoproteus* | MAFUS02 | Yes | Found in hatch year *Dumetella carolinensis* at Rushton |
| *Haemoproteus* | MELMEL07 |  | Found in hatch year *Melospiza melodia* during fall migration |
| *Haemoproteus* | PASILI01 |  | Found in hatch year *Dendroica coronata* during fall migration |
| *Haemoproteus* | SIAMEX01 | Yes | Found in resident species *Cardinalis cardinalis* |
| *Haemoproteus* | SPIARB01 |  | Found in hatch year *Zonotrichia albicollis* during fall migration |
| *Haemoproteus* | SPIPAS02 |  | Found in hatch year *Spizella passerina* during fall migration |
| *Haemoproteus* | TUMIG06 | Yes | Found in hatch year *Hylocichla mustelina* at Rushton |
| *Leucocytozoon* | ACAFLA03 |  | Found in multiple resident species in North America |
| *Leucocytozoon* | BAEBIC07 | Yes | Found in resident species *Poecile carolinensis* |
| *Leucocytozoon* | CARCAR05 | Yes | Found in resident species *Cardinalis cardinalis* |
| *Leucocytozoon* | CATMIN01 | Yes | Found in hatch year *Hylocichla mustelina* at Rushton |
| *Leucocytozoon* | CATMIN05 | Yes | Found in hatch year individuals of multiple species during fall migration |
| *Leucocytozoon* | CATUST09 | Yes | Found in hatch year *Hylocichla mustelina* at Rushton |
| *Leucocytozoon* | CATUST28 |  | Found in hatch year *Catharus ustulatus* during fall migration |
| *Leucocytozoon* | CB1 |  | Found in hatch year individuals of multiple species during fall migration |
| *Leucocytozoon* | CNEORN01 | Yes | Found in resident species *Cardinalis cardinalis* |
| *Leucocytozoon* | COLBF21 |  | Found in multiple resident species in North America |
| *Leucocytozoon* | CYACRI04 |  | Found in *Cyanocitta cristata* which does not migrate outside of temperate North America |
| *Leucocytozoon* | CYACRI05 |  | Found in *Cyanocitta cristata* which does not migrate outside of temperate North America |
| *Leucocytozoon* | DUMCAR01 | Yes | Found in resident species *Cardinalis cardinalis* |
| *Leucocytozoon* | HYLMUS02 | Yes | Found in resident species at Rushton |
| *Leucocytozoon* | PERCAN01 |  | Found in hatch year *Zonotrichia albicollis* during fall migration |
| *Leucocytozoon* | POECAR02 | Yes | Found in resident species *Poecile carolinensis* |
| *Leucocytozoon* | POEHUD01 |  | Found in hatch year *Catharus minimus* during fall migration |
| *Leucocytozoon* | SETSTR01 |  | Found in hatch year *Setophaga striata* during fall migration |
| *Leucocytozoon* | TURMIG05 |  | Found in hatch year *Turdus migratorius* during fall migration |
| *Leucocytozoon* | ZOLEU02 | Yes | Found in hatch year *Zonotrichia albicollis* during fall migration |
| *Plasmodium* | BAEBIC04 | Yes | Found in resident species *Baeolophus bicolor* |
| *Plasmodium* | BT7 |  | Found in hatch year birds of multiple species during fall migration |
| *Plasmodium* | CARCAR10 | Yes | Found in resident species *Cardinalis cardinalis* |
| *Plasmodium* | CATUST05 | Yes | Found in hatch year *Hylocichla mustelina* at Rushton |
| *Plasmodium* | CATUST06 |  | Found in hatch year birds of multiple species during fall migration |
| *Plasmodium* | CYACRI03 |  | Found in hatch year *Pipilo erythropthalmus* during fall migration |
| *Plasmodium* | GEOTRI01 |  | Boyd et al. 2018 found in juvenile *Zonotrichia albicollis* in Canada |
| *Plasmodium* | GEOTRI09 | Yes | Found in resident species *Baeolophus bicolor* |
| *Plasmodium* | LINN1 | Yes | Found in hatch year *Hylocichla mustelina* at Rushton |
| *Plasmodium* | PADOM11 | Yes | Found in hatch year birds of multiple species at Rushton |
| *Plasmodium* | PASCYA01 |  | Found in hatch year *Passerina cyanea* during fall migration |
| *Plasmodium* | RWB01 | Yes | Found in resident species *Baeolophus bicolor* |
| *Plasmodium* | SEIAUR01 | Yes | Found in hatch year birds of multiple species at Rushton |
| *Plasmodium* | SETCOR03 | Yes | Found in resident species *Cardinalis cardinalis* |
| *Plasmodium* | SYAT05 |  | Found in hatch year birds of multiple species during fall migration |
| *Plasmodium* | TACTHA01 |  | Found in hatch year *Melospiza melodia* during fall migration |
| *Plasmodium* | TUMIG03 | Yes | Found in hatch year birds of multiple species at Rushton |
| *Plasmodium* | WW3 |  | Found in hatch year birds of multiple species during fall migration |

**Supplementary Table 2**. Cross correlations between mosquitoes and the bird families Turdidae, Parulidae, and Passerellidae in southeastern Pennsylvania. Shown are analyses for all mosquitoes together, and for seven individual mosquito species that were sampled at least 1,000 times between 2016 and 2022. “*Culex pipens-restuans*” represents mosquitos that could not be identified to either *Culex pipens* or *Culex restuans* due to their similar appearance.

| **Mosquito species** | **Bird family** | **Cross correlation at time lag 0** | **t** | **P-value** |
| --- | --- | --- | --- | --- |
| All mosquitoes | Turdidae | -0.652 | -2.9 | **0.003** |
| All mosquitoes | Parulidae | -0.882 | -4.1 | **0.000** |
| All mosquitoes | Passerellidae | -0.550 | -2.5 | **0.014** |
| *Aedes albopictus* | Turdidae | -0.514 | -2.412 | **0.016** |
| *Aedes albopictus* | Parulidae | -0.324 | -1.518 | 0.129 |
| *Aedes albopictus* | Passerellidae | -0.595 | -2.792 | **0.005** |
| *Aedes japonicus* | Turdidae | -0.776 | -3.640 | **0.000** |
| *Aedes japonicus* | Parulidae | -0.910 | -4.266 | **0.000** |
| *Aedes japonicus* | Passerellidae | -0.848 | -3.977 | **0.000** |
| *Aedes triseriatus* | Turdidae | -0.862 | -4.042 | **0.000** |
| *Aedes triseriatus* | Parulidae | -0.809 | -3.795 | **0.000** |
| *Aedes triseriatus* | Passerellidae | -0.786 | -3.688 | **0.000** |
| *Culex pipiens* | Turdidae | -0.648 | -3.037 | **0.002** |
| *Culex pipiens* | Parulidae | -0.767 | -3.598 | **0.000** |
| *Culex pipiens* | Passerellidae | -0.605 | -2.839 | **0.005** |
| *Culex restuans* | Turdidae | 0.510 | 2.391 | **0.017** |
| *Culex restuans* | Parulidae | 0.214 | 1.003 | 0.316 |
| *Culex restuans* | Passerellidae | 0.384 | 1.802 | 0.072 |
| *Culex pipiens-restuans* | Turdidae | -0.848 | -3.979 | **0.000** |
| *Culex pipiens-restuans* | Parulidae | -0.917 | -4.303 | **0.000** |
| *Culex pipiens-restuans* | Passerellidae | -0.632 | -2.964 | **0.003** |
| *Culex salinarius* | Turdidae | -0.402 | -1.884 | 0.060 |
| *Culex salinarius* | Parulidae | -0.553 | -2.594 | **0.009** |
| *Culex salinarius* | Passerellidae | -0.341 | -1.597 | 0.110 |
